# The Assumptions of the Tea Bag Index and Their Implications: A Reply to Mori 2025

**DOI:** 10.1111/ele.70117

**Published:** 2025-04-18

**Authors:** Judith M. Sarneel, Jeff W. Atkins, Laurent Augusto, Janna M. Barel, Sarah Duddigan, Nicolas Fanin, Mariet M. Hefting, Jonas J. Lembrechts, César Marín, Marshall D. McDaniel, Leonardo Montagnani, Tina Parkhurst, Matteo Petit Bon, Adriano Sofo, Joost A. Keuskamp

**Affiliations:** ^1^ Department of Ecology and Environmental Science Umeå University Umeå Sweden; ^2^ USDA Forest Service, Southern Research Station New Ellenton South Carolina USA; ^3^ INRAE, Bordeaux Sciences Agro UMR 1391 ISPA Villenave d'Ornon Cedex France; ^4^ Ecology & Biodiversity Group, Department of Biology Institute of Environmental Biology, Utrecht University Utrecht the Netherlands; ^5^ Department of Geography and Environmental Science University of Reading Reading UK; ^6^ Systems Ecology, A Life Vrije Universiteit Amsterdam Amsterdam the Netherlands; ^7^ Centro de Investigación e Innovación Para el Cambio Climático (CiiCC) Universidad Santo Tomás Valdivia Chile; ^8^ Department of Agronomy Iowa State University Ames Iowa USA; ^9^ Faculty of Agricultural, Environmental and Food Sciences Free University of Bolzano Bolzano Italy; ^10^ School of Environmental and Conservation Sciences Murdoch University Murdoch Australia; ^11^ Department of Wildland Resources | Quinney College of Natural Resources and Ecology Center Utah State University Logan Utah USA; ^12^ Department of Agricultural, Forestry, Food and Environmental Sciences (DAFE) University of Basilicata Potenza Italy; ^13^ Biont Research Utrecht the Netherlands

## Abstract

Responding to Mori (2025), we discuss that the simplifications and implications of the Tea Bag Index are essential to its ease of use. However, they necessitate careful attention, especially regarding the appropriate incubation time. Aligning with Mori (2025), we call for a deeper understanding of the interpretation of *k_TBI*.

## Graphical abstract

Responding to Mori (2025), we discuss that the TBI's simplifications are essential to its ease of use. However, their implications necessitate careful attention, especially regarding appropriate incubation time. Aligning with Mori (2025), we call for a deeper understanding of the interpretation of k_TBI.

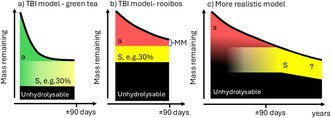

## 1 Introduction

The breakdown of organic material is the result of several processes (e.g., leaching, fragmentation, bleaching, enzymatic hydrolysis) that comprise the process of *decomposition*. The relative importance of these processes can change over time and space, across litter types and material fractions within a given litter type, but they all result in loss of organic material from the original unit (e.g., a leaf). A relatively straightforward and common way to study decomposition of plant material is by determining mass loss curves (Wieder and Lang 1982). However, such mass loss curves are laborious and time‐consuming to obtain and difficult to compare across ecosystems because unstandardised leaf litter is used.

**FIGURE 1:**
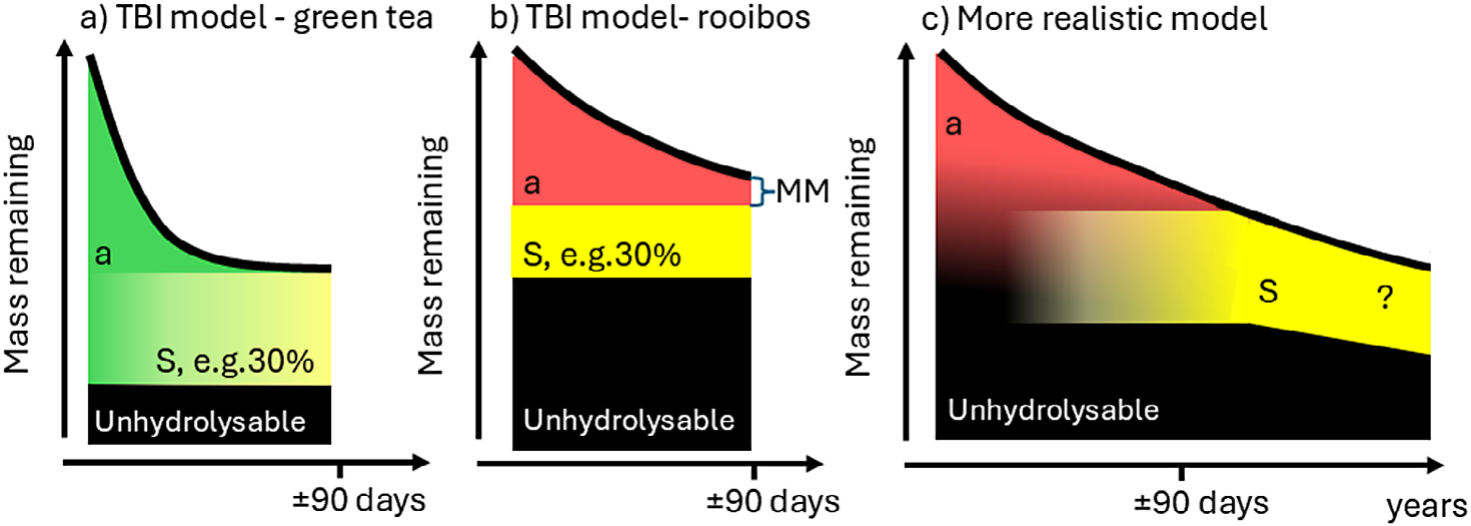
Reasoning of the Tea Bag Index (TBI) model. (a) The TBI is underpinned by a three‐fraction decomposition model, with (1) a labile fraction (a; green or red shading) which drives mass loss during early decomposition, (2) a stabilised fraction (yellow) which is derived from incomplete digested compounds from the hydrolysable fraction and (3) an unhydrolysable recalcitrant fraction (black) parameterised by Soxhlet analysis. Using the unique difference in decomposition dynamics between green tea and rooibos, the formation of the stabilised fraction is derived from green tea mass loss after 90 days. It is scaled to the hydrolysable fraction to obtain the stabilisation factor (*S_TBI*). In the TBI, the mass losses from the yellow and black fractions are assumed to be negligible on the short timescales of three months (Assumption 2). (b) To obtain fraction *a* for rooibos, *S* is scaled to the hydrolysable fraction of rooibos and mathematically assumed to form instantaneously (Assumption 3). The *k_TBI* is subsequently estimated from the observed mass loss of rooibos, provided that rooibos has not yet reached its asymptote (Assumption 1), which can be quantified by calculating a mass margin (MM). (c) TBI is a simplification of the reality, where the difference between the hydrolysable and unhydrolysable fraction is not as strict, unhydrolysable material decomposes from the start and can create rest products that are equivalent to hydrolysable material. Hence, TBI does not predict long‐term decomposition dynamics (Sarneel et al. 2024).

The Tea Bag Index (TBI) was introduced to provide an easy‐to‐quantify and standardised proxy for the decomposition process of plant material during early phases of decomposition (Keuskamp et al. 2013). The method consists of incubating a slow‐decomposing rooibos and a fast‐decomposing green tea as equivalents of mesh bags with local leaf litter. The tea bag types are incubated for 90 days at 8 cm soil depth. Subsequently, the *observed* mass losses of rooibos and green tea are evaluated using a decomposition *model* with three fractions (i.e., decomposable (labile) material, stabilised material, and recalcitrant material). The model is *parameterised* by using the mass loss observed in rooibos and green tea and the hydrolysable fractions of both tea types (Figure 1). Hence, the TBI provides two proxies of decomposition dynamics: initial decomposition rate (*k_TBI*; Box S1) and stabilisation factor (*S_TBI*, Box S1). The *k_TBI* is used to characterise initial mass loss rates of the hydrolysable fraction of rooibos, whereas *S_TBI* is used to characterise the built up of recalcitrant rest material from the hydrolysable fraction.

In a recent analysis of the TBI, Sarneel et al. (2024) showed that, globally, *k_TBI* and *S_TBI* are negatively correlated, although certain environmental conditions (vegetation type and/or climate) can induce deviations from this general trend. In a response to the TBI method in general and to Sarneel et al. (2024) in particular, Mori (2025) pointed out that the correlations between *k_TBI* and *S_TBI* and deviations from the relation reported by Sarneel et al. (2024) should be interpreted cautiously because

Aspect 1: A mathematical dependence of *k_TBI* on *S_TBI* (derived from transferring *S_TBI* from green tea to rooibos) could bias correlation analysis.

Aspect 2: Fundamental differences in decomposition dynamics between both litter types resulting in different responses to environmental conditions could cause deviations from the general negative trend between *k_TBI* and *S_TBI* observed in Sarneel et al. (2024).

Aspect 3: A deviation between *k_TBI* and the observed initial decomposition rate, as derived from mass loss curves of rooibos of ca. 90 days (*k_real*; Box S1).

With this work, we aim to contribute to the discussion on how and when the TBI should or should not be used by first examining its assumptions and subsequently by discussing the aspects raised by Mori (2025). To this end, we expanded the data set of 21 laboratory TBI timeseries incubations of tea used by Mori (2025) with nine unpublished time series (Table S1). With this expanded data set, we followed the procedure outlined by Mori (2025) and calculated *k_TBI* and *Asymptote_TBI* predicted by the TBI at 90 days as well as the observed initial decomposition rate and asymptote (respectively, *k_real* and *Asymptote_real*; Box S1). We related the predicted and observed parameters to each other and investigated what determined the reliability of the prediction (for details on the methodological approach, see Appendix S1). Since Mori's aspects tie to assumptions underlying the TBI, we first discuss these assumptions and conclude by responding to Mori (2025) explicitly.

Assumption 1 The acid unhydrolysable fraction does not decompose within 90 days.

**FIGURE 2:**
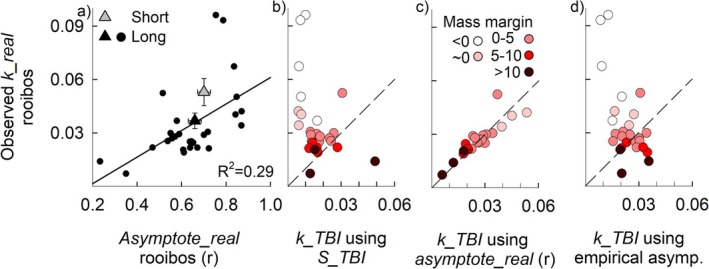
The importance of the asymptote for *k_real*. The observed asymptote (*asymptote_real*) and observed initial decomposition rate (*k_real*) correlate positively in timeseries of rooibos tea (a). Triangles indicate means of the 24 successful fits and show that fits from shorter timeseries (60 days; triangle) result in a higher observed *asymptote_real* and *k_real* compared to the longer timeseries (90–120 days). Error bars are SE. Despite the relationship between the mass loss in green and rooibos (suggesting transferability of stabilisation, Figure S2), *S_TBI* does poor in predicting the observed *k_real* in rooibos (b), especially when mass loss margins (indicated by dot colour) are small. The dashed line indicates the 1:1 line. Small mass margins suggest that rooibos has approached its asymptote at 90 days (Assumption 2; Figure S1), and those observations hence fall outside the TBI framework. Using the observed *asymptote_real* of rooibos to calculate the *k_TBI* highly improves the predictive power (c), suggesting the sensitivity of *k_TBI* to estimation of the asymptote. However, using the empirical relation between the asymptotes of green tea and rooibos (Figure S2; Box S1) to predict the rooibos asymptote does not improve the predictive power of *k_TBI* (d).

Although parts of the lignified fraction can decompose in 90 days, initial decomposition rates are primarily driven by the loss of the labile, hydrolysable material fraction (Hall et al. 2020; Yi et al. 2023). Yet, the distinction between fractions may be less strict in practice, as partial digestion of the recalcitrant fraction can generate hydrolysable compounds (Aswin et al. 2024). Therefore, decomposition rates derived from timeseries data (TBI or otherwise) integrate mass loss rates of different fractions and processes. Consequently, higher decomposition rates can be observed in shorter time series (Figure 2a), as they primarily capture the rapid loss of labile material, whereas longer time series increasingly reflect the slower degradation of recalcitrant material. This means that timeseries observations of mass loss of rooibos tea may be incompatible with TBI (that aims to model the hydrolysable fraction) when Assumption 1 does not hold. The violation of Assumption 1 can be recognised by negative *S_TBI* values (suggesting unhydrolyzable fraction decomposition), which was observed in 2.37% of the analysed pixels in Sarneel et al. (2024), with a minimum of *S_TBI* = −0.16 (Figure S3). Although core to the TBI framework, it is often overlooked that longer periods are unsuitable for calculating the TBI decomposition parameters. To prevent too long incubations, Sarneel et al. (2024) restricted incubation duration (45–135 days).

Assumption 2 An incubation of 90 days allows green tea to reach stabilisation (*S_TBI*) and is sufficient for rooibos to reflect initial decomposition rate (*k_TBI*).

The slowly decomposing rooibos and the rapidly decaying green tea differ in their decomposition dynamics (Figure 1). After an incubation of ca. 90 days, the mass loss of rooibos represents the initial decay rates, whereas the mass loss of green tea represents an asymptote (Figure 1a,b). As Mori (2025) points out (Aspect 2) the mass loss of green tea and rooibos differ in their decomposition dynamics. Assumption 2 is needed to interpret this difference but, consequently, an incubation that is too long will result in rooibos reaching its asymptote, whereas green tea may not reach its asymptote when the incubation is too short. The latter will result in overestimation of *k_TBI*, and a positive correlation between *k_TBI* and *S_TBI*. Using the extended time series data, we quantified how close the TBI measurement was in relation to reaching the asymptote in both rooibos and green tea. To this end, we calculated the mass margin (Box S1) for both rooibos and green tea. To align Assumption 2, a TBI measurement would require a small mass margin for green tea and a large one for rooibos. We indeed observed a small mass margin for green tea (on average 0.2% ± 2.7% SD of the initial dry tea mass; Figure S1a) but for rooibos, a larger variation in the mass margin was observed. Here, the differences between the predicted and observed initial decomposition rates decreased with increasing mass margins, suggesting a negligible differences at mass margins > 5%–10% (Figures 2b and S1b). Calculating the mass margin for rooibos (Box S1) can quantify if this assumption is met. We suggest maintaining a mass margin of > 10% for rooibos. Though smaller margins may suffice (Figure 2), 10% provides a clear benchmark while remaining measurable in terms of mass loss precision.

In conclusion, the standard TBI incubation duration is suitable for green tea to reach its asymptote, yet it appears to be unsuitable to reliably calculate *k_TBI* on certain occasions (Figures S5 and S6). However, in the data set of Sarneel et al. (2024), we could not find clear environmental conditions that would more frequently result in violation of this assumption (i.e., mass margin rooibos < 10%; Figure S7). We reproduced the results of Sarneel et al. (2024) by including only rooibos measurements that likely met Assumption 2 (with mass margins > 10%; including 66.4% of the tea bag incubations). This showed that, despite some changes in the absolute range of *k_TBI*, the overall patterns remained the same (Figures S5 and S6). However, given that the 90 days may be too long in one third of the considered measurements, a careful consideration of the incubation duration is needed (Box S1).

Assumption 3 The stabilisation factor scales with the hydrolysable fraction and can hence be transferred across litter types.

The literature on ‘limit factors’ suggests that stabilisation factors may scale with the chemical composition of the leaf material, since limit factors bear conceptual similarity to *S_TBI*. Nevertheless, Mori (2025) suggested that this is not the case (Aspect 3), since the asymptote predicted by the *S_TBI* does not correlate 1:1 with the observed *asymptote_real* in the rooibos time series. However, the significant relationship between the observed *asymptote_real* of green tea and rooibos (*F*_1,25_ = 39.0; *p* < 0.001; *R* ^2^ = 0.59) suggests that stabilisation factors of rooibos and green tea do scale. The divergence from the 1:1 line could be due to the TBI parameterisation, although using the parameterisation by Hayes et al. (2024; Figure S3) did not improve the predictions. Alternatively, it could be that the stabilisation of green tea may not scale identically with stabilisation in rooibos. When using the observed *asymptote_real* of rooibos instead of *S_TBI*, a 1:1 correlation between the observed *k_real* and *k_TBI* was found (Figure 2c; Box S1). This suggests a high sensitivity of *k_TBI* to asymptote estimations. As an alternative way to predict the asymptote of rooibos (Mori, pers. commun.), we used the empirical relationship between the remaining mass fraction of green tea (the asymptote of green tea in TBI) and the *asymptote_real* of rooibos (*F*_1,25_ = 29.8; *p* < 0.001; *R* ^2^ = 0.54; Figure S2). This, however, did not increase the predictive power of *k_TBI* (Figure 2d).

While this analysis supports the assumption that *S_TBI* is a parameter that can be transferred across litter types, it also shows that (1) transferring *S_TBI* underpredicts the asymptote of rooibos and that (2) the determination of the initial decomposition rate is sensitive to the asymptote. Nonetheless, rooibos may reach its asymptote on timescales where the recalcitrant material will also start contributing more significantly to mass loss (see Assumption 1). This impairs the estimation of a stabilisation factor for rooibos and hampers comparisons of predicted and observed parameters. Further, *S_TBI* is mathematically implemented at the start of the decomposition, ignoring that at the incubation time when the TBI is calculated, stabilisation may not be completed yet. This would lead to faster *k_TBI* compared to *k_real*. In the time series, however, *k_TBI* underpredicts *k_real* (Figure 2a), making it unlikely that this is a major issue. Last, it should be stressed that transferring *k_TBI* across litter types is outside the scope of the TBI, as that would imply that all litter types would approach the asymptote at the same time.

## 2 Response to Mori (2025), Conclusions and Perspectives

The TBI aims to provide an easily applicable method to gain insight into short‐term decomposition dynamics of labile litter fractions and highlights that initial decomposition rates and asymptotes are separate characteristics of the decomposition process. The TBI, however, does not intend to elucidate the dynamics of labile versus recalcitrant litter types nor does it try to obtain site‐specific insights in decomposition dynamics of local litter. The strength of the TBI lies in its simple application and its standardisation across time and space. This allowed Sarneel et al. (2024) to describe global patterns of *k_TBI* and *S_TBI* across environmental gradients. By analysing the relationships between each parameter and environmental conditions separately, Sarneel et al. showed an overall negative correlation as well as deviations from this relationship. Mori raised that a mathematical dependence (Aspect 1) as well as observations derived from different litters (Aspect 2) could contribute to these patterns. However, the observed relationship between *asymptote_real* and *observed k_real* (Figure 2a) corroborates that those parameters can be independently influenced by environmental conditions. In addition, as outlined by Mori et al. (2022), the mathematical dependence between *k_TBI* and *S_TBI* would induce a positive correlation, whereas Sarneel et al. (2024) observed that environmental conditions induced a negative correlation between *k_TBI* and *S_TBI*. Aligning with Mori's (2025) Aspect 3, we show that *k_TBI* often provides a poor estimation of *k_real*. We suggest that maintaining a mass margin (Assumption 2) could alleviate this issue, but our data set was too small to support this statistically. We further show that the mismatch between *k_TBI* and *k_real* derives from the high sensitivity of *k_TBI* (but likely *k* values in general) to the estimation of the asymptote.

In conclusion, the ecological interpretation of *k_TBI* calls for further investigation as it does not necessarily reflect the mass loss rates observed in rooibos tea. Whether it reflects the mass loss rate of hydrolysable material (as it intends to do) would require advanced chemical analysis. Yet, comparing litter types could benefit from considering which process drove the observed mass loss, especially since the initial decomposition rate and the asymptote are likely influenced by different environmental factors. As showcased in Sarneel et al. (2024) and here, separating these parameters can provide valuable insights into decomposition.

## Author Contributions

J.M.S. wrote the original draft with significant input from all other coauthors.

### Peer Review

The peer review history for this article is available at https://www.webofscience.com/api/gateway/wos/peer‐review/10.1111/ele.70117.

## Supporting information


Data S1.

## Data Availability

The data added to the data described in Mori (2025) is available on zenodo https://zenodo.org/records/15083202.
