# Supplementary material for: The Assumptions of the Tea Bag Index and Their Implications: A Reply to Mori 2025
Source: Ecol Lett. 2025 Apr 18;28(4):e70117. doi: 10.1111/ele.70117 (PMC12006831; doi:10.1111/ele.70117)
Supplement: Supplementary file 1 — Data S1. [file ELE-28-0-s001.docx]

**Appendix to: The assumptions of the Tea Bag Index and their implications; A reply to Mori 2024**

Judith M. Sarneel, Jeff W. Atkins, Laurent Augusto, Janna M. Barel, Sarah Duddigan, Nicolas Fanin, Mariet M. Hefting, Jonas J. Lembrechts, César Marín, Marshall D. McDaniel, Leonardo Montagnani, Tina Parkhurst, Matteo Petit Bon, Adriano Sofo, Joost A. Keuskamp

**Index**

**Appendix 1: Extended method and analysis on the timeseries dataset**

**Appendix 2: Re-evaluating the data of Sarneel *et al.* (2024) when keeping a large mass margin for rooibos tea**

**Appendix 3: Box S1; list of terms**

**Appendix 1: Extended method and analysis on the timeseries dataset**

The used dataset consists of 32 timeseries of mass losses observed for rooibos and green tea under different conditions (Table S1). The dataset consists mainly of tea from nonwoven bags, but for 18 time series, the tea was placed in hand made woven bags. Given this heterogeneity, we included all bag types in the analysis.

For each tea type at each condition, we fitted a logistics regression and extracted the observed initial decomposition rate (*k_real*; consistent with the terminology in Mori, 2024) as well as the observed asymptote (*asymptote_real*). We calculated the Tea Bag Index (TBI) for the datapoint that was closest to 90 days. We used Nonlinear Least Squares (R version 4.3.1) to fit the mass fraction remaining by a*e^-^*^k^*^*^*^t^*+ (1-*a*), where *a* is the observed asymptote (*asymptote_real*), *k* is the observed initial decomposition rate (*k_real*) during the time period of the observation (*t;* days). The TBI proxies were fitted following Keuskamp *et al.* (2013), on the timestep closest to 90 days (Table S1).

The relation between the observed *asymptote_real* in rooibos and green tea was tested using a linear model with *asymptote_real* in rooibos as the dependent parameter in R version 4.3.1 (R Core Team 2023). Likewise, the relation between the TBI asymptote of green tea (equalling the mass remaining of green tea at 90 days) and *asymptote_real* in rooibos was determined.

To quantify the sensitivity of *k_real* and *asymptote_real* to the duration of the incubation series, we calculated those proxies on the full time series (90-120 days) as well on time series where we removed all measurements that were longer than 60 days. We averaged *k_real* and *asymptote_real* across all timeseries where fits were obtained in both the longer and short time series (Figure 2a).

For each mass remaining at 90 days, for both the timeseries dataset described here as well as the dataset used by Sarneel *et al.* (2024), we used the mass for rooibos tea observed at the time when *k_TBI* was calculated to estimate how close this observation is to the asymptote (mass margin; Box 1). For the time series dataset, we used the observed asymptote as derived from our nonlinear least squares analysis, and for the dataset in Sarneel *et al.* (2024), we used *S_TBI* to estimate the asymptote for rooibos. In addition, we calculated the difference between *asymptote_TBI* and *asymptote_real* of green tea and between *k_TBI* and *k_real*, to visualize how the mass margin related to the predictive power of the *TBI_S* and *TBI_k*, respectively.

Next to *k_TBI* (Keuskamp et al. 2013), we calculated two other initial decomposition rates using the rooibos mass remaining at ca. 90 days and following the TBI formula’s. However, we substituted the asymptote based on *S_TBI* by 1) the observed *asymptote_real* in rooibos, and 2) by an estimated asymptote based on the empirical relation between green tea mass remaining (which equals the asymptote of green tea in the TBI model) and *asymptote_real* in rooibos. The empirical relation between green tea mass remaining and *asymptote real* is described as asymptote_rooibos = 1.135 * mass remaining green + 0.233.

We also explored the role of parameterization by calculating *k_TBI* and *S_TBI* using the hydrolysable fractions described in Hayes *et al.* (2024), with *H* = 0.7967 and 0.6352 for green tea and rooibos, respectively

**Table S1**: Overview of the dataset presented in Fig 2 and 3. The table indicates the source, a general description of the incubation conditions, the type of tea used (with W for woven, NW for non-woven, PB for plant based, and W/NW tea from nonwoven bags placed in woven bags), if the initial mass of the bags was determined by oven drying (at ca 60/70C) or by subtracting a correction for moisture from the initial mass, the number of replicates per timestep (*n*), the number of timesteps included, and for which duration (in days) the *k_TBI* and *S_TBI* were calculated and lastly, an indication for which of the timeseries the model could not be fitted in R or the calculation of the TBI proxies failed. Sources are 1: Duddigan *et al.* (2020), 2: Keuskamp *et al.* (2013), 3: Mori (2022b), 4: Mori (2022a), 5: Middelanis *et al.* (2023) and 6: Sarneel (unpublished).

| **Source** | **General conditions** | **Mesh** | **initial** | **n** | **Time steps** | **TBI duration** | **Failure** |
| --- | --- | --- | --- | --- | --- | --- | --- |
| 1 | 20 C, fertile soil | W | dried | 3 | 14 | 91 |  |
| 2 | 15 degrees, forest soil | W | none | 6 | 7 | 68 |  |
| 2 | 25 degrees, forest soil | W | none | 6 | 7 | 68 |  |
| 3 | Unknown | W/NW * | dried | 1 | 7 | 90 |  |
| 3 | Unknown | W/NW * | dried | 1 | 7 | 90 |  |
| 3 | Unknown | W/NW * | dried | 1 | 7 | 90 |  |
| 3 | Unknown | W/NW * | dried | 1 | 7 | 90 |  |
| 3 | Unknown | W/NW * | dried | 1 | 7 | 90 |  |
| 3 | Unknown | W/NW * | dried | 1 | 7 | 90 |  |
| 3 | Unknown | W/NW * | dried | 1 | 7 | 90 |  |
| 3 | Unknown | W/NW * | dried | 1 | 7 | 90 |  |
| 3 | Unknown | W/NW * | dried | 1 | 7 | 90 |  |
| 3 | Unknown | W/NW * | dried | 1 | 7 | 90 |  |
| 4 | 3C, moist | W/NW * | dried | 1 | 6 | 90 |  |
| 4 | 25 C, dry | W/NW * | dried | 1 | 6 | 90 | fit, k_TBI |
| 4 | 3C, moist | W/NW * | dried | 1 | 6 | 90 |  |
| 4 | 25 C, dry | W/NW * | dried | 1 | 6 | 90 |  |
| 4 | 3C, moist | W/NW * | dried | 1 | 6 | 90 |  |
| 4 | 25 C, dry | W/NW * | dried | 1 | 6 | 90 | fit |
| 4 | 3C, moist | W/NW * | dried | 1 | 6 | 90 |  |
| 4 | 25 C, dry | W/NW * | dried | 1 | 6 | 90 | fit |
| 5 | 14 C, moist | W | dried | 3 | 4 | 90 | fit |
| 5 | 14 C, moist | NW | dried | 3 | 4 | 90 | fit, k_TBI |
| 6 | 10 C, moist potting soil | NW | none | 5 | 5 | 99 |  |
| 6 | 20 C, moist potting soil | NW | none | 5 | 5 | 99 |  |
| 6 | 10 C, dry potting soil | NW | none | 5 | 5 | 99 |  |
| 6 | 20 C, dry potting soil | NW | none | 5 | 5 | 99 |  |
| 6 | 18C, potting soil | PB | none | 2-4 | 5 | 90 |  |
| 6 | 18C, potting soil | PB | dried | 2-4 | 5 | 90 |  |
| 6 | 30 C, moist soil/sand | NW | none | 8 | 6 | 84 |  |
| 6 | 15 C, moist soil/sand | NW | none | 8 | 4 | 84 |  |
| 6 | 2 C, moist soil/sand | NW | none | 8 | 5 | 84 |  |


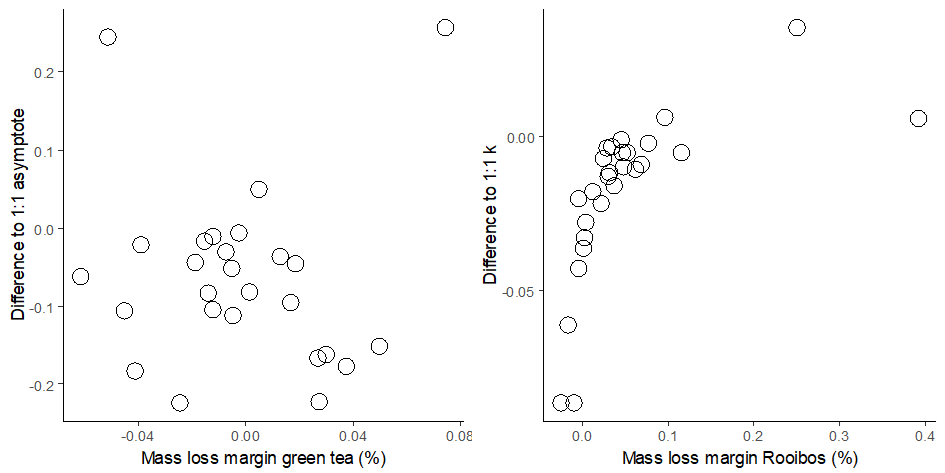


b)

a)

Figure S1: Deviations from the 1:1 line of the TBI-predicted and observed asymptotes of green tea (residues) in relation to the mass margin of green tea (a). In (b) absolute differences between predicted and observed initial decomposition rate of rooibos tea (residues) are given showing in relation to the mass margin of rooibos indicating how far the decomposition curve has approached the asymptote of rooibos tea. In other words, the mass margin indicates whether assumption 2 is violated or not. Because the relation is not linear in b) visual inspection suggest that the mass margin of rooibos requires to be around 10% to predict the observed *k_real*. Note that the scale of a and b are different orders of magnitude.

Figure S2: Empirical estimation of the asymptote of rooibos based on the relation between the green tea asymptote as described by TBI (equalling the mass remaining at 90 days) and the observed asymptote in rooibos. The relation is described by asymptote rooibos = 1.135 * asymptote TBI of green tea + 0.233, where the asymptote of green tea is defined by the mass fraction remaining in green tea after 90 days, which, given the small mass margin of green tea is a valid assumption.

**Effect of parametrization on the relation between predicted and observed decomposition dynamics.**

Figure S3: Using a different parameterization of the unhydrolysable fraction of rooibos and green tea does not change the mismatch observed between calculated and observed asymptote (a) and initial decomposition rate (b) of rooibos tea. The predicted asymptote was calculated following the procedure described in Keuskamp *et al.* (2013), either using the original hydrolysable fraction (white dots; H = 0.842 and 0.552 for green tea and rooibos respectively) or using the hydrolysable fraction described in Hayes *et al.* (2024), with H = 0.7967 and 0.6352 for green tea and rooibos respectively. Each point represents one timeseries and the red line indicates the 1:1 line, while the black lines indicate fitted regression lines.

**Appendix 2: Re-evaluating the data of Sarneel *et al.* (2024) when keeping a large mass margin for rooibos tea**

Since analyses in Appendix 1 suggest that a mass margin of ca 10% is needed for *TBI_k* to reflect the observed *k_real*, we re-ran the analyses presented in Sarneel *et al.* (2024) using only the data where the mass margin for rooibos is >10% (taking into account S_TBI). Removing these observations slightly changed the range of observed values (Figure S3). We explored if there were consistent differences between the two categories of measurements and show both the original figures as well as the updated figures of our re-analysis. In general, we did not find systematic differences that could explain conditions under which the mass margin becomes too small (Figure S4). Re-analysis of the patterns described by Sarneel *et al.* (2024) decreased the absolute values of *k_TBI*, but the patterns across large gradients remained the same (Figure S5, S6).

*
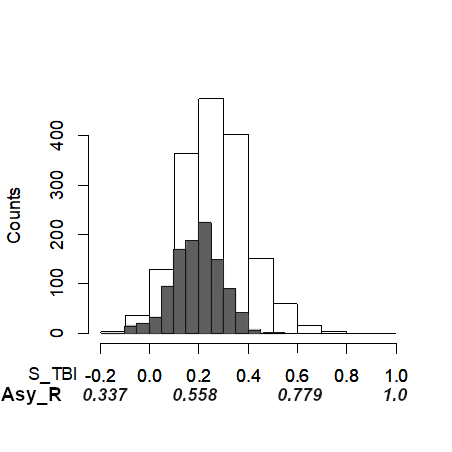
*

Figure S4: Distribution of S_TBI values in the original dataset of Sarneel *et al.* (2024) indicated with white bars and in the dataset where all the observations with a mass margin of less than 10% in rooibos have been removed (dark shaded bars). On the x-axis, the *S_TBI* values are indicated as well as the associated predicted asymptote of rooibos tea (*Asy_R*) based on this *S_TBI.*

Figure S5: Mean *k_TBI* and *S_TBI* per biome as presented in a) Sarneel *et al.* (2024) and b) when removing measurements with a mass margin smaller than 10% in rooibos. “Colour coding follows main climatic conditions, with red for tropical, orange for temperate, blue for cold, black for wetlands and white for dry ecosystems. Forest biomes are indicated by triangles and low vegetation system by circles. Values shown are corrected for spatial autocorrelation” as described in Sarneel *et al.* (2024). Error bars are standard errors. Biome names follow Olson *et al.* (2001) with abbreviations as in Sarneel *et al.* (2024).


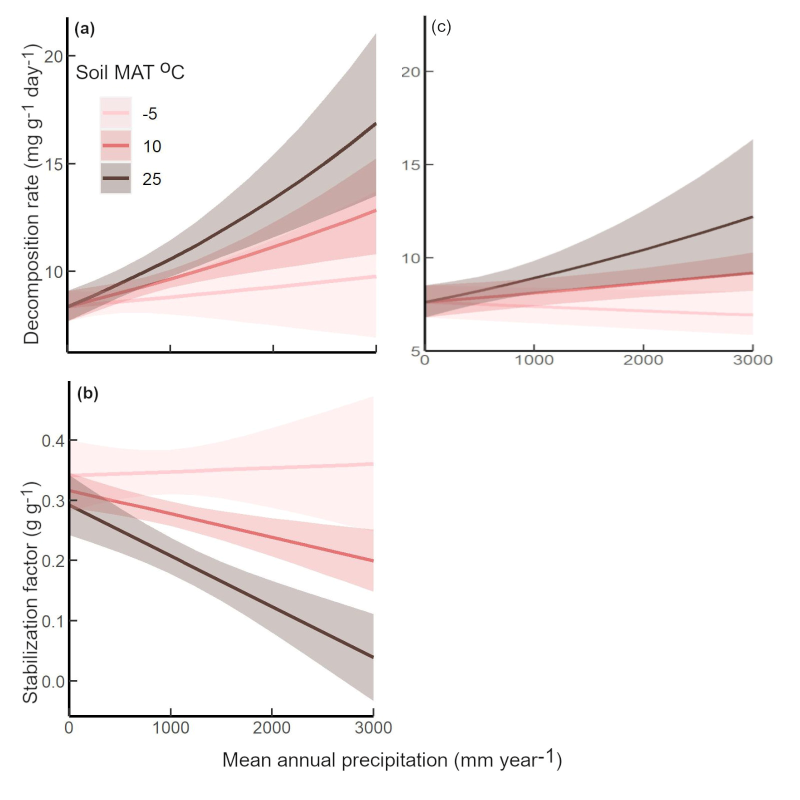


Figure S6: The interaction of both *k_TBI* and *S_TBI* with Mean Annual soil Temperature (MAYT) and Mean Annual Precipitation (MAP) causes decoupling in dryer and colder environments. Relationship between mean annual precipitation (MAP) and *k_TBI* (a) *S_TBI* (b) for different values of mean annual soil temperature, based on the models described in Sarneel *et al.* (2024) and c) for the re-analysis taking only measurements with a large mass margin into account (>10%). Lines indicate the mean and the shaded areas the confidence intervals obtained using ‘predictSE.gls’ in the AICcmodavg package in R.


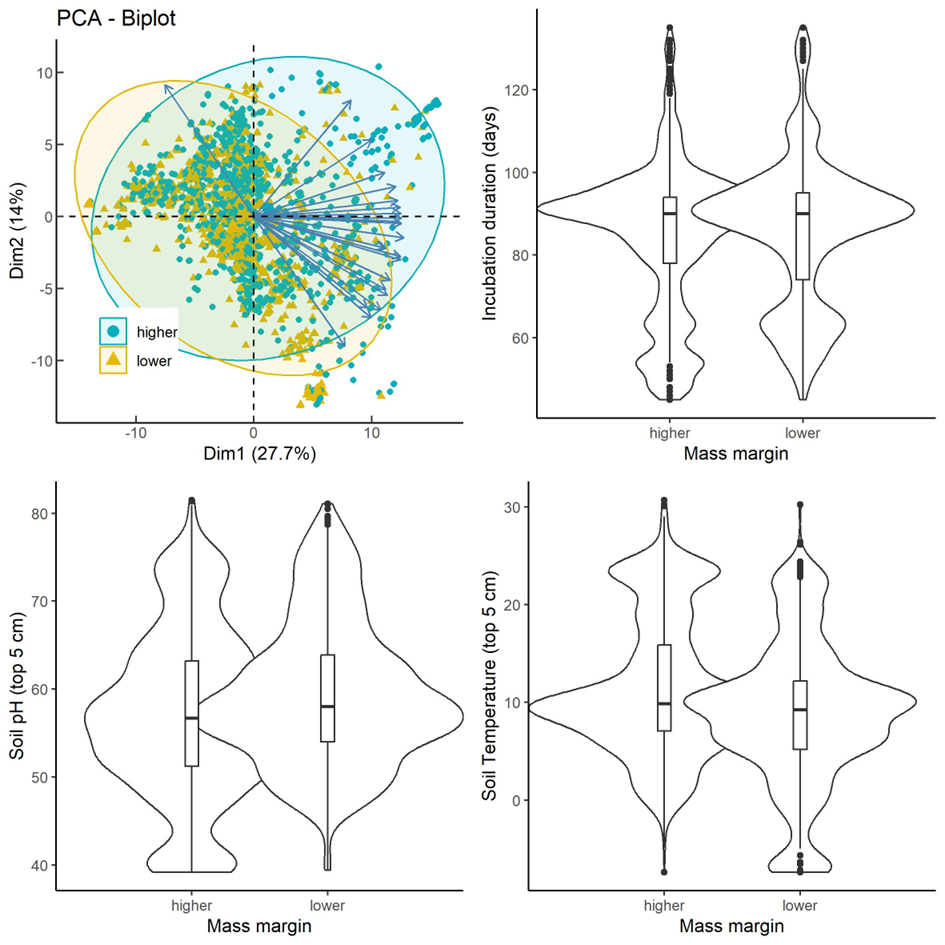


d

c

b

a

Figure S7: a) PCA plot (See Sarneel *et al.* 2024 for a description of the parameterspace) with yellow points indicating measurements where the mass margin of rooibos was <10% (lower), and blue points for measurements with a mass margin larger than 10% (higher). The distribution and median for b) incubation duration, c) Soil pH in the top 5 cm based on water extractions and derived from <https://www.soilgrids.org> as well as d) mean annual soil temperature derived from Lembrechts *et al.* (2022). The analysis in Figure S4 and S5 are based on the data in the ‘higher’ category.

Box S1 **List of terms**

**Asymptote_TBI**: For rooibos this encompasses the unhydrolyzable fraction plus the stabilized fraction of the hydrolysable fraction (H) rooibos. For green tea, the *asymptote_TBI* equals the mass fraction remaining at 90 days. Conceptually representing (1-a) in eq. 2 and calculated by using the stabilization factor (*S_TBI*):

| $\boldsymbol{Asymptote\_TBI}_{\boldsymbol{(rooibos)}}=0.552*S\_TBI+0.448$ | eq. 1 |
| --- | --- |

**Asymptote_real:** Observed asymptote in timeseries. That is, the measured mass fraction at which the mass loss curve is observed to level off. It can be observed for green tea and rooibos separately. Conceptually representing (1-a) in eq. 2.

***Asymptote_empirical****:* Predicted asymptote of rooibos as derived from the relation between mass fraction remaining of green tea and the observed *asymptote_real* of rooibos tea given by: *asymptote_empirical* = 1.135 * *asymptote_TBI* green tea + 0.233, where *asymptote_TBI* green tea is defined by the mass fraction remaining at the time point of measuring.

**Decomposition model**: The Tea Bag Index (TBI) is based on the decomposition model for early-stage decomposition of fresh litter described by Wieder and Lang (1982), which characterizes an exponential decay towards an asymptote (eq. 2), reflecting varying decomposition rates among material fractions. As the TBI focuses on the initial phases of decomposition, mass loss is predominantly driven by the rapid decomposition of the most labile fraction, while the rates for more recalcitrant fractions are minimal

| $M_{t}=ae^{-kt}+\left( 1-a \right)$ | eq. 2 |
| --- | --- |

**Hydrolysable fraction (H):** Fraction of fresh litter that are dissolved in the Soxhlet procedure (*H* green tea= 0.842 ; *H* rooibos = 0.552 , Keuskamp *et al.*, 2013). This fraction is interpreted as potentially hydrolysable (labile) and comprises the labile and the stabilized fraction.

***k_TBI*:** Decomposition rate estimated following the TBI method, describing the mass loss dynamics of the labile fraction following eq. 2. In short, it is calculated at 90 days of incubation by scaling S_TBI and resolving eq 2. using the observed mass loss of rooibos. Equivalent to k1_TBI_ (Sarneel *et al*. 2024). For this work, we recalculated *k_TBI*, once by using the *asymptote_real* of rooibos and second by using *Asymptote_empirical*, by substituting those for (1-a) in eq 2.

***k_real*:** Observed decomposition rate derived from time series (Appendix 1) using Nonlinear Least Squares models.

**Labile fraction (*a*)**: Easy to decompose material. Described by decomposition rate constant (*k*). For TBI, *a* is quantified by the mass loss fraction observed in green tea after 90 days.

**Mass margin (MM)**: The difference between the observed mass fraction remaining at 90 days and observed *asymptote_real*, Indication how far decomposition has advanced to the asymptote. Can be calculated for both rooibos and green tea time series as well as field data on rooibos tea when replacing *asymptote_real* by its best approximation (*asymptote_TBI*):

| $\boldsymbol{Mass margin}=Mt-Asymptote\_real rooibos =Mt-Asymptote\_TBI$ | eq. 3 |
| --- | --- |

**Stabilized fraction:** The part of *H* that becomes stabilized and more recalcitrant to decomposition.

**Stabilization factor (*S_TBI*):** The stabilized fraction is scaled to the size of the hydrolysable fraction. *S_TBI* is equivalent to *S_TBI_* (Sarneel *et al*. 2024) and *S* (Keuskamp *et al*. 2013) and quantified as:

| $S\_TBI=1- \frac{a_{g}}{H_{g}}$ | eq. 4 |
| --- | --- |

**Unhydrolyzable, or acid-insoluble, fraction**: The fraction of fresh litter that is not soluble in fat, water or acid and is left after the Soxhlet extraction (green tea: 0.258; rooibos: 0.448; Keuskamp et al., 2013). This material is considered to be recalcitrant to decomposition and contains a high proportion of lignified material.

**References**

1.

Duddigan, S., Shaw, L.J., Alexander, P.D. & Collins, C.D. (2020). Chemical Underpinning of the Tea Bag Index: An Examination of the Decomposition of Tea Leaves. *Applied and Environmental Soil Science*, 2020.

2.

Hayes, E.B., Norris, C.E. & Volpe, J.P. (2024). A field assessment to validate the assumptions of the Tea Bag Index (TBI) as a measure of soil health. *Applied Soil Ecology*, 195.

3.

Keuskamp, J.A., Dingemans, B.J.J., Lehtinen, T., Sarneel, J.M. & Hefting, M.M. (2013). Tea Bag Index: a novel approach to collect uniform decomposition data across ecosystems. *Methods in Ecology and Evolution*, 4, 1070-1075.

4.

Lembrechts, J.J., van den Hoogen, J., Aalto, J., Ashcroft, M.B., De Frenne, P., Kemppinen, J. *et al.* (2022). Global maps of soil temperature. *Global Change Biol.*, 28, 3110-3144.

5.

Middelanis, T., Pohl, C.M., Looschelders, D. & Hamer, U. (2023). New directions for the Tea Bag Index: Alternative teabags and concepts can advance citizen science. *Ecol. Res.*, 38, 690-699.

6.

Mori, T. (2022a). Is the Tea Bag Index (TBI) Useful for Comparing Decomposition Rates among Soils? *Ecologies*, 3, 521-529.

7.

Mori, T. (2022b). Validation of the Tea Bag Index as a standard approach for assessing organic matter decomposition: A laboratory incubation experiment. *Ecol. Indicators*, 141.

8.

Olson, D.M., Dinerstein, E., Wikramanayake, E.D., Burgess, N.D., Powell, G.V.N., Underwood, E.C. *et al.* (2001). Terrestrial ecoregions of the worlds: A new map of life on Earth. *Bioscience*, 51, 933-938.

9.

R Core Team (2023). R: A language and environment for statistical computing. R Foundation for Statistical Computing Vienna, Austria.

10.

Sarneel, J.M., Hefting, M.M., Sandén, T., van den Hoogen, J., Routh, D., Adhikari, B.S. *et al.* (2024). Reading tea leaves worldwide: Decoupled drivers of initial litter decomposition mass-loss rate and stabilization. *Ecol. Lett.*, 27.
